# Downregulation of miRNA-638 promotes angiogenesis and growth of hepatocellular carcinoma by targeting VEGF

**DOI:** 10.18632/oncotarget.8930

**Published:** 2016-04-22

**Authors:** Jiwen Cheng, Yanke Chen, Pu Zhao, Xi Liu, Jian Dong, Jianhui Li, Chen Huang, Rongqian Wu, Yi Lv

**Affiliations:** ^1^ Department of Hepatobiliary Surgery, Institute of Advanced Surgical Technology and Engineering, First Affiliated Hospital of Xi'an Jiaotong University, Xi’ an, Shaanxi, China; ^2^ Department of Pediatric Surgery, Second Affiliated Hospital of Xi'an Jiaotong University, Xi'an, Shaanxi, China; ^3^ Department of Genetics and Cell Biology, Environment and Genes Related to Diseases Key Laboratory of Education Ministry, College of Medicine, Xi'an Jiaotong University, Xi'an, Shaanxi, China; ^4^ Department of Neonatology, Shaanxi Provincial People's Hospital, Xi'an, Shaanxi, China; ^5^ Department of Pathology, First Affiliated Hospital of Xi'an Jiaotong University, Xi'an, Shaanxi, China; ^6^ Department of Surgical Oncology, Shaanxi Provincial People's Hospital, Xi'an, Shaanxi, China

**Keywords:** hepatocellular carcinoma, miR-638, vascular endothelial growth factor, angiogenesis, prognosis

## Abstract

The expression and function of microRNA-638 (miR-638) in hepatocellular carcinoma (HCC) remained unknown. Using the miRNA target prediction tools, we predicted that the vascular endothelial growth factor (VEGF) might be a direct target of miR-638. The aim of this study was to test the hypothesis that downregulation of miRNA-638 promotes angiogenesis and growth of HCC by targeting the VEGF signaling pathway. We found that miR-638 was significantly downregulated in HCC cells and clinical HCC specimens, and miR-638 levels were inversely correlated with tumor size, portal vein invasion and poor prognosis. Overexpression of miR-638 inhibited the processes of tumor angiogenesis *in vitro* and *in vivo*. The xenograft mouse model experiments showed miR-638 repressed tumor growth of HCC *in vivo*. Using a luciferase reporter assay, we identified VEGF as a direct target of miR-638. Subsequent investigation revealed that miR-638 expression was inversely correlated with VEGF expression in human HCC samples. Taken together, these results suggested that miR-638 is a novel therapeutic target for HCC and overexpression of miR-638 could suppress angiogenesis and tumor growth of HCC by inhibiting VEGF signaling.

## INTRODUCTION

Active angiogenesis and metastasis are responsible for rapid recurrence and poor survival of patients with hepatocellular carcinoma (HCC) [1]. Identification of the key players in these processes may provide promising breakthroughs for improving the prognosis of these patients. MicroRNAs (miRNAs) have been recognized as important post-transcriptional regulators in normal liver physiology and liver diseases [2–5]. Recently, dysregulation of miR-638 has been described in several different types of human tumors. But its role in tumorigenesis remains controversial. For instance, miR-638 was significantly downregulated and may play a cancer suppressing role in human gastric cancer [6], basal cell carcinoma [7], breast cancer [8], non-small cell lung cancer [9], colorectal carcinoma [10], and chronic lymphocytic leukemia [11]. However, it has been shown to promote tumorigenesis in human colon carcinoma HCT-116 cells and human osteosarcoma U2OS cells [12] and to enhance proliferation of esophageal squamous carcinoma cells *in vitro* [13]. Moreover, miR-638 promotes melanoma metastasis, protects melanoma cells from apoptosis and autophagy [14], and aggravate DNA damage in the benzo(a)pyrene-induced carcinogenesis [15]. A recent study reported that the expression of miR-638 was higher in liver cancer tissues than normal liver tissues [16]. However, in colorectal liver metastases, miR-638 has been shown to be significantly downregulated [17]. Although the above evidence suggests that miR-638 may play an important role in tumorigenesis and tumor progression, the expression and functional role of miR-638 in HCC remained largely unknown. Using the miRNA target prediction tools, we predicted that the vascular endothelial growth factor (VEGF) might be a direct target of miR-638. The VEGF signaling pathway is critical in the angiogenic process [18]. Increased expression of VEGF and its receptors has been associated with poor prognosis in HCC [19, 20]. We therefore hypothesized that downregulation of miRNA-638 promotes angiogenesis and growth of hepatocellular carcinoma by targeting the VEGF signaling pathway. The current study was intended to test this hypothesis.

## RESULTS

### miR-638 is downregulated in HCC

**Figure 1:**
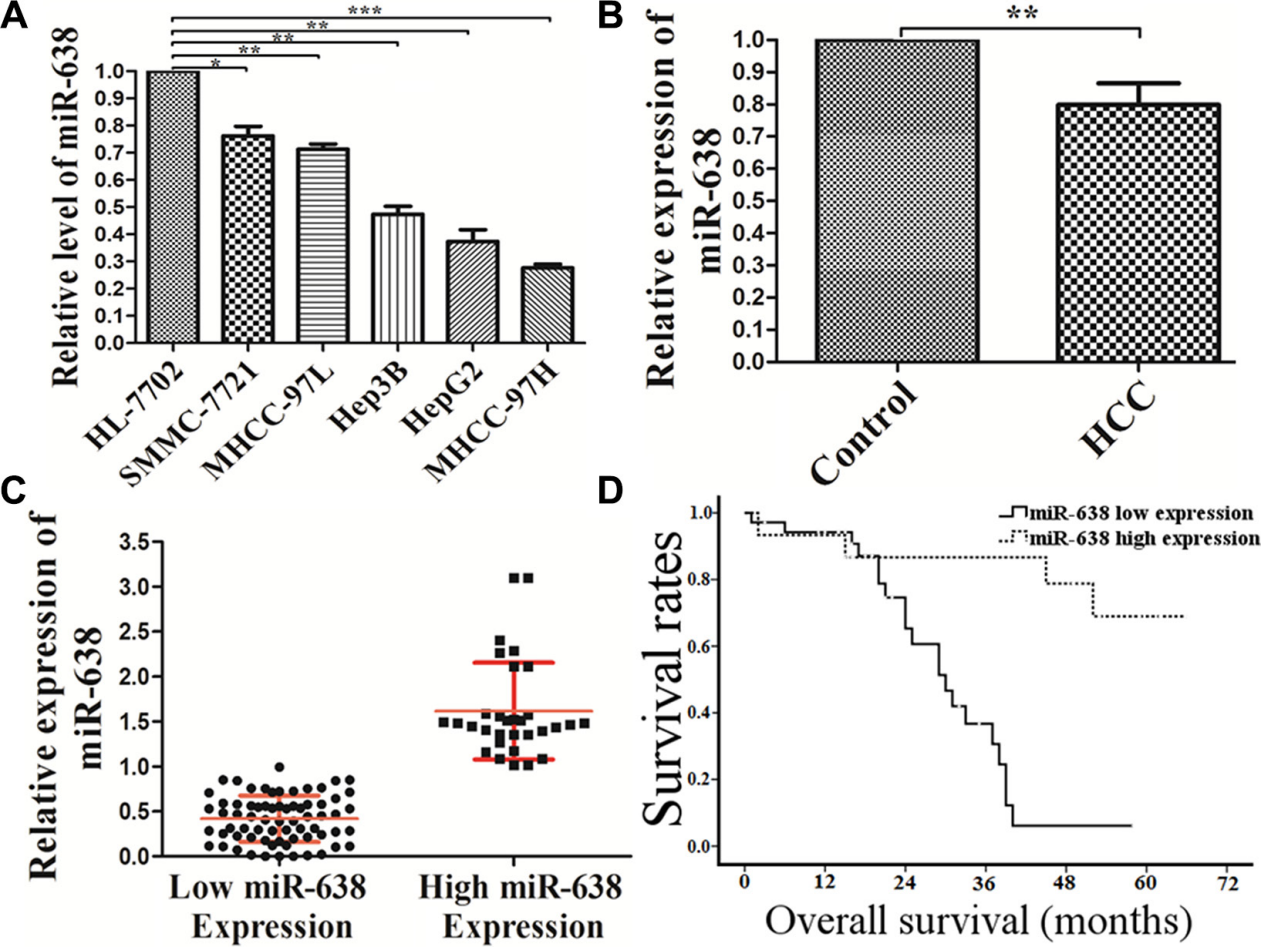
The expression of miR-638 was reduced in HCC cell lines and clinical specimens (**A**) qRT-PCR analysis of miR-638 expression in human normal hepatic cell line HL-7702 cells and HCC cell lines (MHCC-97L, HepG2, Hep3B, SMMC-7721 and MHCC-97H). (**B**) The average expression level of miR-638 in 100 HCC tissue samples compared with non-cancerous samples. (**C**) The average expression level of miR-638 in the high miR-638 expression group compared with the low miR-638 expression group. (**D**) The correlation between miR-638 expression and overall survival (OS) of the patients after surgery. Curves of OS were plotted by Kaplan-Meier methods according to miR-638 expression levels. HCC: hepatocellular carcinoma tissues. *P < 0.05, **P < 0.01, ***P < 0.001.

To explore the role of miR-638 in the development and progression of HCC, miR-638 expression was examined in HCC cell lines and clinical HCC specimens. As shown in Figure 1A, miR-638 levels were significantly lower in all 5 HCC cell lines (MHCC-97L, HepG2, Hep3B, SMMC-7721 and MHCC-97H) as compared with normal liver cells (*P < 0.01, P < 0.01, P < 0.01, P < 0.05, P < 0.001*, respectively). Similarly, 69% of clinical HCC samples have lower levels of miR-638 expression in tumor tissues than those in adjacent non-tumor tissues from the same patient (*P < 0.01*, Figure 1B). Samples with lower levels of miR-638 expression in tumor tissues than those in adjacent non-tumor tissues were considered miR-638 low-expression. And samples with higher levels of miR-638 expression in tumor tissues than those in adjacent non-tumor tissues were considered miR-638 high-expression. The relative miR-638 expression levels of the high group (*n* = 31) and the low group (*n* = 69) were shown in Figure 1C. HCC patients in the miR-638 low-expression group had a median survival period of 21.23 months, whereas HCC patients in the miR-638 high-expression group had a median survival period of 50.33 months (Figure 1D). The difference in the median survival period between miR-638 low- and high-expression groups was statistically significant (*P < 0.001*). In addition, the relative miR-638 expression levels were inversely correlated with tumor size and portal vein invasion (*P = 0.016, P = 0.012*, respectively, Table 1).

**Table 1:** The analysis of relationship between the relative expression level of miR-638 and clinicopathological feature in HCC

Clinicopathological feature	Number of cases (*n* = 100)	miR-638 expression level		*p* value (chi-squared)
		High (31)	Low (69)	
**Age (years)**				0.829
< 50	43	14	29	
≥ 50	57	17	40	
**Gender**				0.300
Women	22	9	13	
Men	78	22	56	
**HBV**				0.092
+	73	19	54	
−	27	12	15	
**Cirrhosis**				0.640
+	70	23	47	
−	30	8	22	
**Tumor Size (cm)**				***0.016***
≤ 5	37	17	20	
> 5	63	14	49	
**AFP (ug/L)**				0.108
≤ 20	33	14	19	
> 20	67	17	50	
**TNM Stage**				0.072
I + II	35	15	20	
III + IV	65	16	49	
**Portal vein invasion**				***0.012***
Yes	35	5	30	
No	65	26	39	
**Lymph node metastasis**				0.598
Yes	20	5	15	
No	80	26	54	

### VEGF is a direct target of miR-638 in HCC

**Figure 2:**
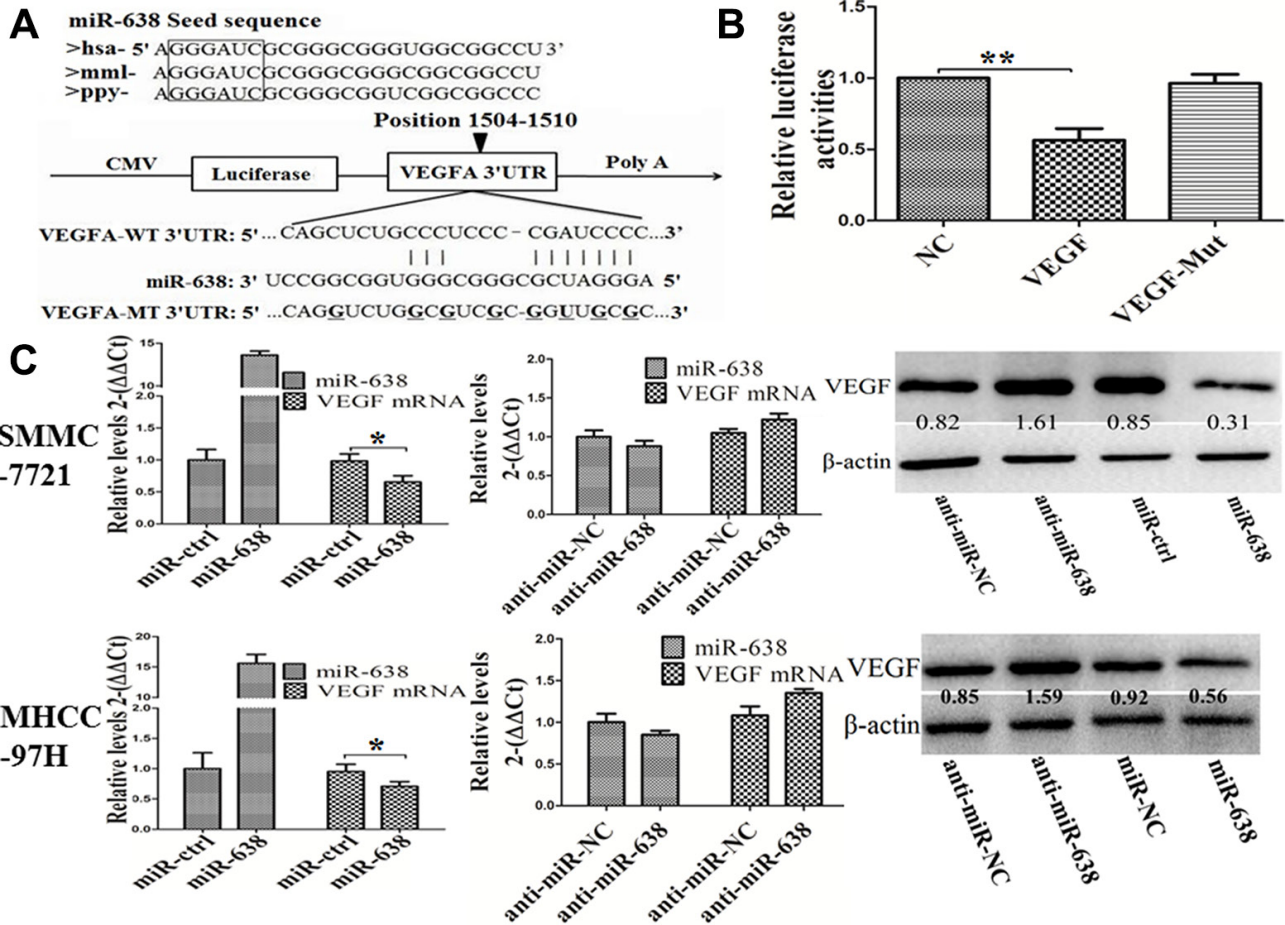
VEGF is a direct target of miR-638 in HCC (**A**) Conserved miR-638 seed region sequence in the 3′-UTR of VEGF among different species. (**B**) luciferase assay in HEK293 cells. pLUC-VEGF vector or pLUC-VEGF mut vector was co-transfected with miR-638. The relative luciferase activities were measured 24 h post-transfection and normalized by calculating the ratio of firefly luciferase to Renilla luciferase activity. (**C**) mRNA and protein expression level of VEGF were measured by qRT-PCR and Western blot analyses in SMMC-7721 and MHCC-97H. The ratios of detected VEGF against β-actin were shown under each Western blot image. Data represented the mean ± SEM of three independent experiments. *P < 0.05, **P < 0.01.

**Figure 3:**
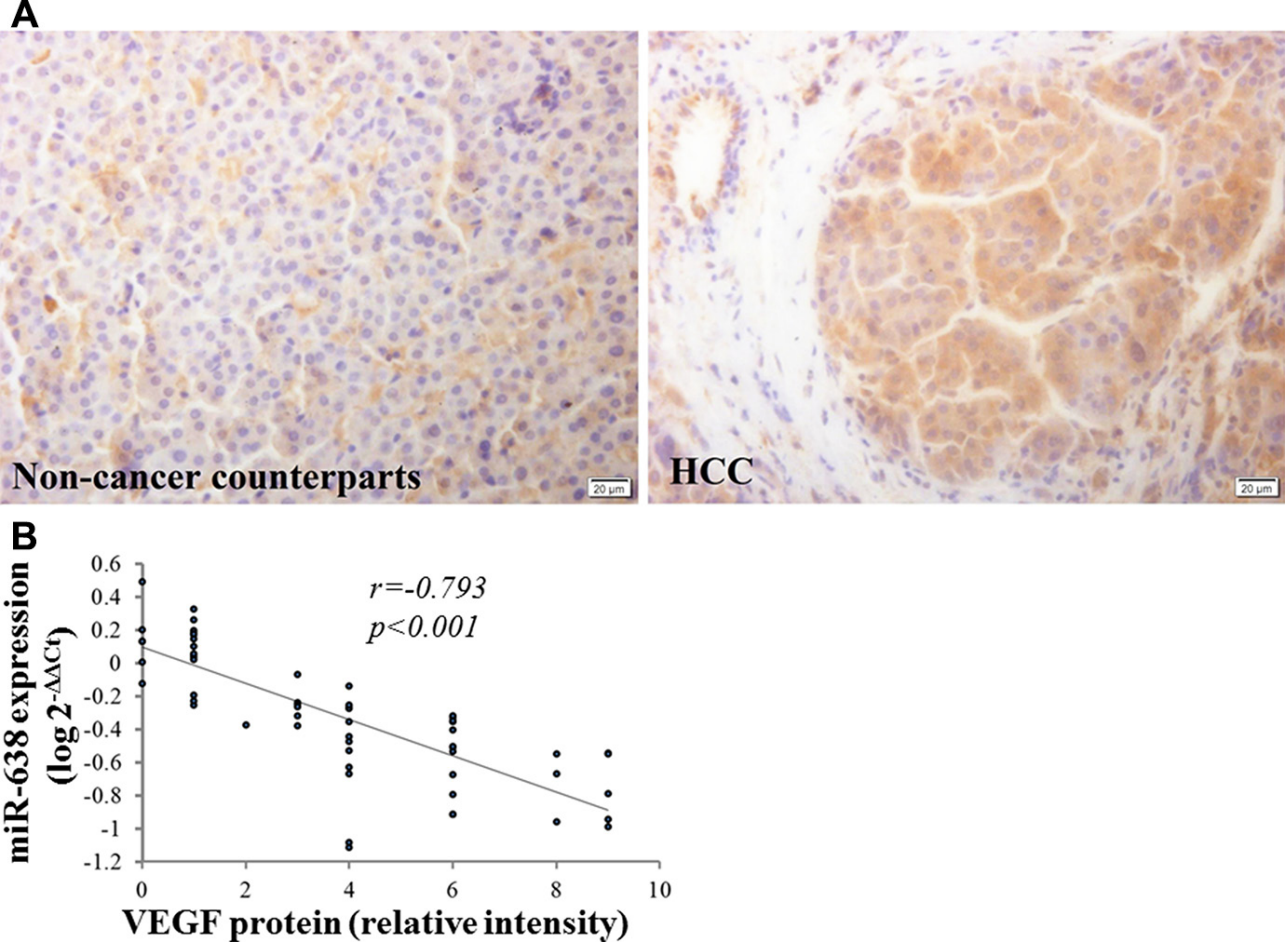
Inverse correlation between miR-638 and VEGF expression in HCC tissues (**A**) The protein expression levels of VEGF were analyzed by immunohistochemistry staining (40×). (**B**) Inverse correlation between miR-638 and VEGF expression in HCC tissues. Statistical analysis was performed using Pearson's correlation coefficient. A logarithmic transformation has been used in the Spearman's rank correlation coefficient test. HCC: hepatocellular carcinoma tissues.

To uncover the mechanisms by which miR-638 affects the malignant phenotype of HCC, we searched for its potential target genes in the TargetScan (http://www.targetscan.org/), PicTar (http://pictar.mdc-berlin.de/) Microrna.org (http://www.microrna.org/) and RegRNA (http://regrna.mbc.nctu.edu.tw) databases. We found that VEGF was one of the top candidates. As shown in Figure 2A, the region complementary to the miR-638 seed region was found in the 3′-UTR of human VEGF. To validate whether VEGF was a direct target gene of miR-638, a luciferase reporter assay was conducted. As shown in Figure 2B, overexpression of miR-638 significantly decreased the luciferase activity of wild-type VEGF but not that of mutant VEGF. Furthermore, miR-638 overexpression significantly reduced VEGF mRNA and protein expression in both SMMC-7721 (Figure 2C upper) and MHCC-97H cells (Figure 2C lower). To further investigate the relationship between VEGF and miR-638, we examined the expression of VEGF and miR-638 in 60 FFPE HCC specimens. We found that clinical HCC samples have lower levels of VEGF protein in tumor tissues than those in adjacent non-tumor tissues from the same patient (Figure 3A), and the VEGF protein levels miR-638 levels were inversely correlated with VEGF levels (*r* = −*0.79*3, P < 0.001, Figure 3B). Taken together, the above data suggest that miR-638 directly suppress VEGF expression in HCC.

### miR-638 inhibits tumor angiogenesis *in vitro* and *in vivo*

**Figure 4:**
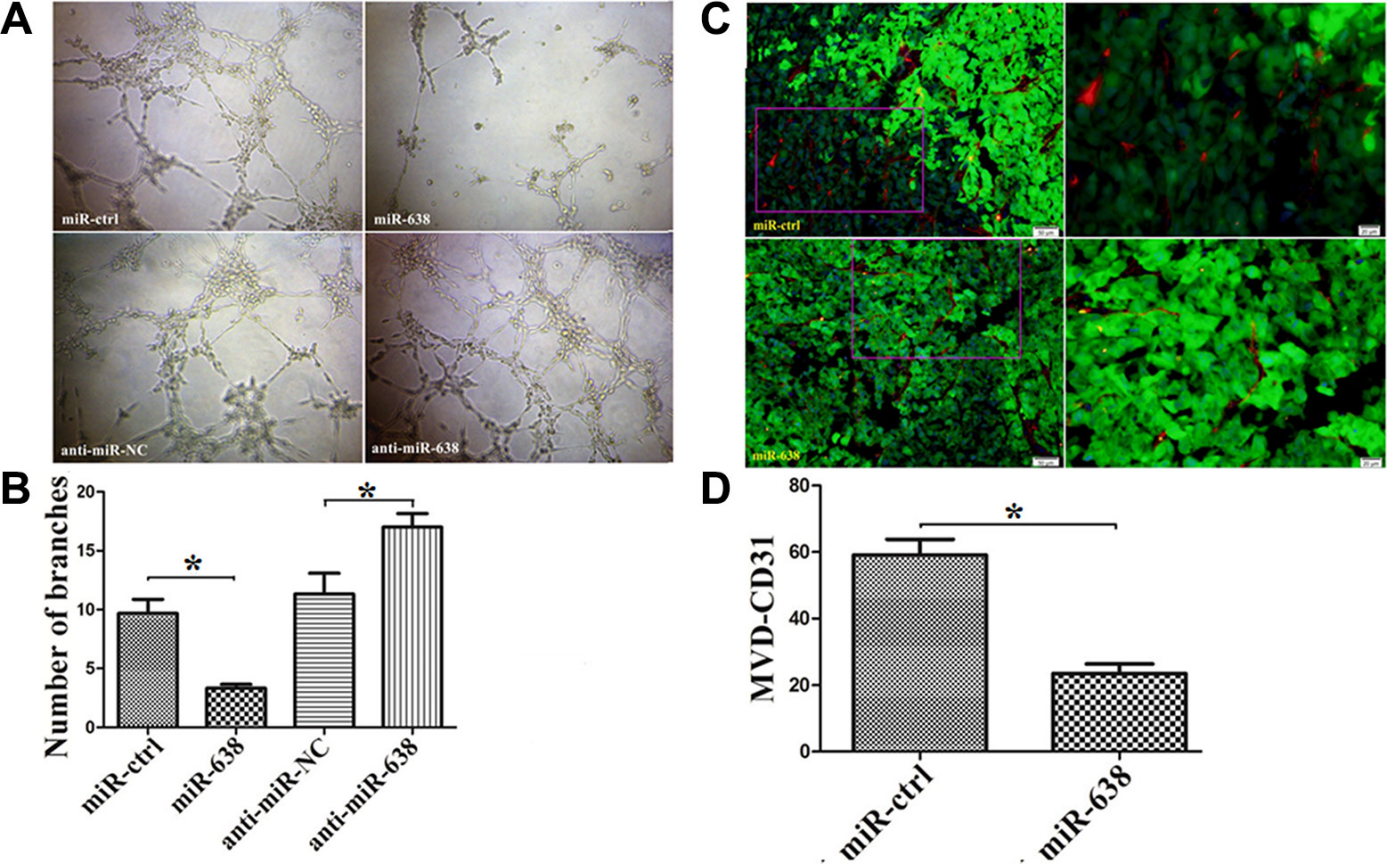
miR-638 inhibits tumor angiogenesis *in vitro* and *in vivo* Angiogenesis *in vitro* and *in vivo* was assessed by the measurement of the tube formation in HUVECs and the microvessel density (MVD) in the mouse tumor xenograft tissues, respectively. (**A**) Representative pictures of the HCC cell-promoted HUVECs tube formation in cells treated with miR-ctrl, miR-638, anti-miR-NC, anti-miR-638. (**B**) Quantification of the branches formed by HUVEC cells. (**C**) Representative pictures of MVD in the mouse tumor xenograft tissues. MVD was measured by immunofluorescence staining of CD31. Red: CD31, Green: SMMC-7721 cells (**D**) Quantification of the levels of MVD in the mouse tumor xenograft tissues. *P < 0.05.

To confirm that miR-638 is a potential angiogenesis suppressor in HCC, we investigated the influence of miR-638 on the angiogenesis both *in vitro* and *in vivo*. Tube-formation assays with HUVECs were performed with the different group of conditioned medium (anti-miR-638, pre-miR-638 or empty vectors). Compared with control cells, ectopic expression of miR-638 by pre-miR-638 vectors dramatically repressed the tube-forming capacity of HUVECs, whereas suppressing endogenous miR-638 by anti-miR-638 vectors promoted the tube-forming capacity of HUVECs (Figure 4A, 4B). Microvessel Density (MVD) is one of the most commonly used parameters to assess the degree of neovessels formation in the tumor tissues. In this regard, the level of MVD was quantified in a mouse tumor xenograft by an indirect immunofluorescence assay of CD31. As shown in Figure 4C and 4D, the amount of MVD in the miR-638 group was remarkably lower than that in the control group. These results suggest that miR-638 represses tumor angiogenesis in HCC.

### miR-638 inhibits hepatocellular carcinoma tumor growth *in vivo*

**Figure 5:**
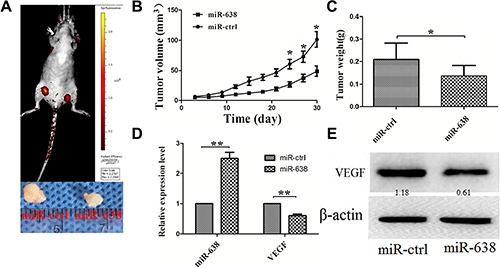
miR-638 inhibits HCC progression *in vivo* miR-638 and control vector-transfected SMMC-7721 cells were injected subcutaneously into different posterior flanks of the same nude mice. The mice were observed for xenograft growth for 4 weeks. (**A**) The tumor volume was assessed *in situ* by a small animal imaging analysis four weeks after tumor inoculation (left flank: ctrl-transfected; right flank: miR-638-transfected). The lower portion of the figure showed the gross morphology of tumors at four weeks after tumor inoculation. (**B**) Tumor growth curves. (**C**) Tumor weight. (**D**) The levels of miR-638 and VEGF mRNA in the tumor tissues from the animals. (**E**) The protein levels of VEGF in the tumor tissues from the animals. *P < 0.05, **P < 0.01.

To assess the effects of miR-638 on tumor growth *in vivo*, miR-638 and control vector-transfected SMMC-7721 cells were injected subcutaneously into different posterior flanks of the same nude mice. The mice were observed for xenograft growth for 4 weeks. As shown in Figure 5A, tumor growth was significantly suppressed by LV-miR-638 as compared with the LV-miR-ctrl. This trend was also confirmed by the sizes and weights of tumors excised from the animals. At day 30, the average volume of LV-miR-ctrl infected tumors was significantly larger than that of LV-miR-638 infected tumors (Figure 5B). Similarly, the average tumor weight of LV-miR-ctrl infected tumors was remarkably heavier than that of LV-miR-638 infected tumors at day 30 (Figure 5C). Furthermore, the expression of miR-638 was increased and the expression of the VEGF protein was reduced in miR-638-treated tumors (Figure 5D and 5E). These data indicate that miR-638 is capable of inhibiting tumor growth and VEGF expression *in vivo*.

## DISCUSSION

Dysregulation of miR-638 has been described in several different types of human tumors, including human gastric cancer [6], basal cell carcinoma [7], breast cancer [8], nonsmall-cell lung cancer [9], colorectal carcinoma [10] and chronic lymphocytic leukemia [11]. A recent study showed that miR-638 expression was upregulated in HCC tissues as compared with normal liver tissues [16]. However, the sample size in that study was very small: 5 normal liver samples versus 20 HCC samples. In the current study, we assessed the miR-638 expression in a relatively large sample sizes (100 HCC samples). And we used their paired non-cancerous tissues as controls, therefore minimized individual variations. We found that the majority of HCC patients, 69 out of 100, has lower levels of miR-638 in cancer tissues than adjacent non-cancerous tissues. Consistent with the results observed in clinical HCC samples, significantly lower expression of miR-638 was observed in several HCC cell lines than normal human hepatic cells. Furthermore, we found that low miR-638 expression was correlated with poor prognosis of HCC patients. The above results strongly indicated that miR-638 is involved in the progress of HCC, and may serve as a novel biomarker for the prognosis of HCC. These findings were in line with several previous studies in some other human tumors. Downregulation of miR-638 in colorectal cancer predicts poor survival [10]. miR-638 was one of four miRNAs identified from genome-wide serum miRNA profiling that predict survival in nasopharyngeal carcinoma patients [21].

Overexpression of miR-638 significantly suppresses the growth of gastric cancer cells [6], while downregulation of miR-638 promotes proliferation and invasion of human NSCLC cells [9] and colorectal carcinoma cells [10]. However, how miR-638 regulates cancer cells growth remains elusive. Angiogenesis is a fundamental factor in tumor growth and metastases. In this study, both *in vitro* and *in vivo* data indicated that miR-638 is capable of suppressing HCC angiogenesis. Overexpression of miR-638 significantly inhibited tumor growth and reduced angiogenesis in a mouse tumor xenograft model. VEGF is a key regulator of angiogenesis. Numerous studies have demonstrated that increased VEGF expression is related to high proliferative index, portal vein tumor thrombosis and poor prognosis in HCC patients [22, 23]. Inhibiting VEGF has been shown to repress tumor angiogenesis and HCC growth under both *in vitro* and *in vivo* conditions [24]. Using four different miRNA target gene prediction databases, we found that VEGF is one of the potential direct target genes for miR-638. This prediction was confirmed by a luciferase reporter analysis. Moreover, our current study showed that overexpression of miR-638 in SMMC-7721 and MHCC-97H cells decreased VEGF mRNA and protein expression. Most mircoRNAs act as rheostats to make fine-scale adjustments to protein output [25]. Their repression on the production of their target proteins is typically relatively mild [26]. Here we also showed that a very high copy number of miRNA-638 is required to produce a modest reduction of VEGF expression in cultured HCC cells. But that does not mean miRNA-638's repression on VEGF production is not significant. As our *in vivo* study indicated that overexpression of miR-638 in the mouse tumor xenografts led to reduced VEGF mRNA and protein expression as compared with the control xenografts without miR-638 overexpression. More importantly, we found that miR-638 was inversely correlated with VEGF protein expression in clinical HCC samples. These results indicate that miR-638 plays an important role in the development and progression of HCC by directly suppressing VEGF.

Hepatitis B viral (HBV) load has been recognized as a significant risk factor for hepatocellular carcinoma recurrence and poor prognosis of patients with HBV-related HCC [27–29]. A recent study showed that miR-638 mimics can significantly inhibit HBV replication [30]. In this regard, overexpression of miR-638 may repress the HCC growth and inhibit HBV replication simultaneously. In addition, several other cancer-related genes have been suggested as direct targets of miR638. For instance, miR-638 inhibits cell proliferation by inhibiting Sp2 in gastric cancer [6]. In NSCLC, downregulation of miR-638 has been shown to promote cell proliferation and invasion and to induce mesenchymal-like transition through a SOX2-dependent pathway [9]. miR-638 also inhibits cell proliferation, invasion and regulates cell cycle by suppressing TSPAN1 in human colorectal carcinoma [10]. However, whether these cancer-related genes are also direct targets of miR-638 in HCC remains unknown. Further studies are needed to determine the relationship between VEGF and these genes.

In conclusion, our findings showed that miR-638 is downregulated and inversely correlated with tumor size, portal vein invasion and poor prognosis in HCC. Ectopic expression of miR-638 can repress tumor growth and inhibit angiogenesis by downregulating VEGF. Thus, miR-638 may be a new prognosis marker and a potential target for HCC treatment.

## MATERIALS AND METHODS

### Cell line and human tissue specimens

Human liver cancer cell lines (SMMC-7721, HepG2, Hep3B, MHCC-97L, and MHCC-97H) and the normal human liver cell line HL-7702 cells were cultured in Dulbecco's modified Eagle medium (DMEM, Hyclone, USA) and 1640 medium (1640; PAA Laboratories GmbH) containing 10% fetal bovine serum (FBS, Gibco BRL, USA). One-hundred paired hepatocellular carcinoma and adjacent non-tumor liver tissues, including 60 paired formalin-fixed paraffin-embedded (FFPE) tissues were selected from January 2008 to June 2010 at Department of Pathology, and 40 paired fresh frozen HCC tissues were collected from patients undergoing resection of hepatocellular carcinoma at the First Affiliated Hospital of Xi'an Jiaotong University from August 2013 to June 2014. The relevant characteristics of the studied subjects were shown in Table 1. No patient received local or systemic therapies before surgery and both tumor and matched adjacent non-tumor tissue were histologically confirmed by two independent pathologists. The study was approved by the Ethics Committee of Xi'an Jiaotong University and informed consent was obtained from all patients and voluntary participation prior to the study. The relevant clinicopathological parameters of the studied subjects were collected from patient records.

### RNA extraction, cDNA synthesis, and quantitative real-time PCR

Total RNA was extracted from human FFPE tissues with E.Z.N.A.^®^ FFPE RNA Kit (Omega Bio-Tek, USA), and total RNA was extracted from prepared liver samples and cultured cells with TRizol reagent (Invitrogen) and cDNA was synthesized following the manufacturer's protocol (Takara). Quantitative real-time PCR (qRT-PCR) analyses were conducted with Power SYBR Green (Takara) according to the manufacturer's instructions. qRT-PCR reactions were performed in the Roche Light Cycler 480 Real-Time PCR Machine. Results were normalized to the expression of U6 or GAPDH and were calculated with the 2^−ΔΔCt^ method [31]. The primers were as follows:

miR-638-RT:5′-GTCGTATCCAGTGCGTGTCGTGGAGTCGGCAATTGCACTGGATACGACAGGCCGC-3′; miR-638-F:5′-ATCCAGTGCGTG TCGTG-3′; miR-638-R:5′-TGCTAGGGATCGCGGGCGGGTG-3′; U6-F:5′-GCTTCGGCAGCACATATACTAAAAT-3′; U6-R:5′-CGCTTCACGAATTTGCGTGTCAT-3′; VEGF-F:TG CAGATTATGCGGATCAAACC; VEGF-R:TGCATTCAC ATTTGTTGTGCTGTAG;GAPDG-F:TGAAGGTCGGA GTCAACGGATT; GAPDH-R:CCTGGAAGATGGTGA TGGGATT;

### Expression vector construction

The miR-638 expression vector (pre-miR-638) and control vector were constructed with synthetic oligonucleotides and cloned in between the 5′ EcoRI and 3′ HindIII sites of the pcDNA6.2-GW/EmGFP vector (Invitrogen) according to the manufacturer's instructions. We also commercially synthesized 2′-O-methyl-modified antisense oligonucleotide of miR-638 was used as miR-638 inhibitor (named anti-miR-638). The sequence of anti-miR-638: 5′-AGGCCGCCACCCGCCCGCGATCCCT-3′. The sequence of negative control (anti-miR-NC) was: 5′-TGACTGTACTGAACTCGACTG-3′. The 3′ UTR of human VEGFA mRNA was constructed by synthetic oligonucleotides and cloned in between the SacI and XhoI sites of the pmirGLO Dual-Luciferase miRNA Target Expression Vector (Promega).

### Dual-luciferase reporter gene assay

Dual-luciferase reporter gene assay was constructed using the Dual-Luciferase Reporter Assay System (Promega) to measure the reporter activity according to the manufacturer's protocol. HEK293 cells of 90% confluence were seeded in 96-well plates. For VEGF 3′-untranslated region (UTR) luciferase reporter assay, 100 ng of wild-type or mutant reporter constructs (pGL3 cm-VEGF-3′UTR-WT or pGL3 cm-VEGF-3′UTR-MUT) were co-transfected into HEK293 cells in 96-well plates with 100 nmol/L miR-638 or 100 nmol/L miR-NC expression vector and Renilla plasmid by using lipofectamine 2000 (Invitrogen). Reporter gene assays were performed 24 hour after transfection. Each assay was repeated in triplicate.

### Western blot

Proteins were separated by sodium dodecyl sulfate-polyacrylamide gel electrophoresis (SDS-PAGE) and transferred to nitrocellulose membrane (Millipore, Bedford, MA, USA). The membranes were blocked with 5% non-fat milk in Tris buffered saline solution with 0.1% Tween-20 (TBST) for 1 hour at room temperature and incubated overnight with rabbit anti-human VEGF and rabbit anti-humanβ-actin primary antibodies at 4°C. VEGF and β-actin antibodies were diluted 1:400 and 1:1000 in TBST containing 1% BSA, respectively. The membranes were washed four times in TBST and incubated with goat anti-rabbit IgG HRP (1:2000; Santa Cruz, CA, USA) for 1 hour at room temperature. The antigen-antibody complex was detected with Immobilon Western HRP Substrate (Merck, Darmstadt, Germany, 500 ml).

### Immunohistochemical analysis

The FFPE tissue samples were sectioned at 5 μm thickness. Sections were deparaffinized with xylene and hydrated using graded alcohol, antigen retrieval and blocking were then performed, and slides were incubated in the primary antibodies (VEGF) at 4°C overnight, followed by incubation with secondary antibodies. Detection was performed by 3, 3′-diaminobenzidine (DAB) and hematoxylin. The level of VEGF protein expression was determined by a semiquantitative scoring method described by Axiotis et al. [32] with minor modifications. Briefly, percentage of tumor cells staining and intensity of immunostaining were assessed as follows: percentage of tumor cells staining: 0, 0%–10%; 1 +, 11%–25%; 2 +, 26%–50%; 3 +, 51%–75%; 4+, 76%-100%; and intensity of immunostaining: 1 +, weak staining; 2 +, moderate staining; 3 +, strong staining. The VEGF protein expression (relative intensity) was calculated by multiplying the percentage of positively stained tumor cells to the staining intensity.

### Immunofluorescence staining

Mouse tumor xenograft specimens were collected at 4 weeks after inoculation in optimum cutting temperature compound and snap-frozen in liquid nitrogen. The frozen tumor xenograft tissues were sectioned into 5 μm pieces, fixed in ice-cold acetone, and were incubated in the rat anti-mouse CD31 primary antibodies (1:200; BD Biosciences, USA) at 4°C overnight, then the cells were rinsed with PBS 3 times and incubated with fluorescent labeled secondary antibody (Alexa flour 488 donkey anti-rabbit IgG (H + L), Thermo Fisher Scientific, Waltham, MA, Country) for 60 min at 37°C. The nuclei were stained with DAPI. The cells were examined with fluorescence microscopy.

### *In vitro* HUVEC tube network formation assay

The tube formation assay was done using HUVECs in the tumor cell–conditioned medium (serum-free conditioned medium from high or low miR-638 expression group and control group). HUVECs (3 × 10^4^) seeded onto 96-well plate coated with Matrigel (BD Biosciences, USA) in conditioned medium at 37°C. Tube formation was found to be optimal after 4–8 h. Tube formation was observed, photographed and quantified under a light microscope.

### *In vivo* tumor xenograft model

Five-week-old female nude mice (BALB/c-nude) were used to analyze tumorigenicity. 1 × 10^6^ SMMC-7721 cells infected with LV-miR-638 and LV-miR-ctrl were suspended in 100 mL PBS and then injected subcutaneously into either side of the posterior flank of the same female nude mouse. Tumor size was measured every 3 days for 4 weeks. Tumor volume (V) was monitored by measuring the length (L) and width (W) with calipers and calculated with the formula (L × W^2^) × 0.5. For endpoint experiments, the bioluminescence images *in vivo* were obtained by the system of photobiology (Xenogen). All experimental procedures involving animals were in accordance with the Guide for the Care and Use of Laboratory Animals and were performed according to the institutional ethical guidelines for animal experiment.

### Statistical analysis

Each experiment was independently repeated at least 3 times. Data were presented as means ± SEM and performed using SPSS13.0 software (SPSS Inc). The differences between 2 independent groups were analyzed using Student *t* test. The relationship between the expression of miR-638 and clinicopathologic characteristics was conducted with Chi-square test. Spearman's correlation was used to explore the association between miR-638 and VEGF expression in the matched hepatocellular carcinoma tumor specimens. *P* < 0.05 was considered statistically significant.

## Abbreviations

HCC: hepatocellular carcinoma
VEGF: vascular endothelial growth factor
FFPE: formalin-fixed paraffin-embedded
HUVECs: human umbilical vein endothelial cells
MVD: microvessel density
OS: overall survival.
